# Comparison of transcriptomic profiles between HFPO-DA and prototypical PPARα, PPARγ, and cytotoxic agents in wild-type and PPARα knockout mouse hepatocytes

**DOI:** 10.1093/toxsci/kfae045

**Published:** 2024-04-04

**Authors:** Melissa M Heintz, William D Klaren, Alexander W East, Laurie C Haws, Steven R McGreal, Rebecca R Campbell, Chad M Thompson

**Affiliations:** ToxStrategies LLC, Asheville, North Carolina 28801, USA; ToxStrategies LLC, Asheville, North Carolina 28801, USA; ToxStrategies LLC, Asheville, North Carolina 28801, USA; ToxStrategies LLC, Austin, Texas 78731, USA; BioIVT LLC, Kansas City, Kansas 66103, USA; BioIVT LLC, Kansas City, Kansas 66103, USA; ToxStrategies LLC, Katy, Texas 77494, USA

**Keywords:** HFPO-DA (GenX), PFAS, peroxisome proliferator-activated receptor α, (PPARα), mode of action (MOA), hepatocytes, transcriptomics

## Abstract

Recent *in vitro* transcriptomic analyses for the short-chain polyfluoroalkyl substance, HFPO-DA (ammonium, 2,3,3,3-tetrafluoro-2-(heptafluoropropoxy)-propanoate), support conclusions from *in vivo* data that HFPO-DA-mediated liver effects in mice are part of the early key events of the peroxisome proliferator-activated receptor alpha (PPARα) activator-induced rodent hepatocarcinogenesis mode of action (MOA). Transcriptomic responses in HFPO-DA-treated rodent hepatocytes have high concordance with those treated with a PPARα agonist and lack concordance with those treated with PPARγ agonists or cytotoxic agents. To elucidate whether HFPO-DA-mediated transcriptomic responses in mouse liver are PPARα-dependent, additional transcriptomic analyses were conducted on samples from primary PPARα knockout (KO) and wild-type (WT) mouse hepatocytes exposed for 12, 24, or 72 h with various concentrations of HFPO-DA, or well-established agonists of PPARα (GW7647) and PPARγ (rosiglitazone), or cytotoxic agents (acetaminophen or d-galactosamine). Pathway and predicted upstream regulator-level responses were highly concordant between HFPO-DA and GW7647 in WT hepatocytes. A similar pattern was observed in PPARα KO hepatocytes, albeit with a distinct temporal and concentration-dependent delay potentially mediated by compensatory responses. This delay was not observed in PPARα KO hepatocytes exposed to rosiglitazone, acetaminophen, d-galactosamine. The similarity in transcriptomic signaling between HFPO-DA and GW7647 in both the presence and absence of PPARα *in vitro* indicates these compounds share a common MOA.

The liver is a common target of toxicity in rodent studies following oral exposure to per- and polyfluoroalkyl substances (PFAS) (Costello *et al.*, 2022). However, different modes of action (MOAs) have been hypothesized for PFAS-mediated liver effects in rodents, likely due to the diversity in chemical structures across PFAS (eg, interchain linkages, carbon chain length). For example, the MOA for liver lesions in mice exposed to the short-chain PFAS, HFPO-DA (ammonium, 2,3,3,3-tetrafluoro-2-(heptafluoropropoxy)-propanoate; CASRN 62037-80-3), has been evaluated in detail using mechanistic and phenotypic data from *in vivo* rodent studies. The current weight of evidence supports that HFPO-DA-mediated liver effects in mice are part of the early key events of the peroxisome proliferator-activated receptor alpha (PPARα) activator-induced rodent hepatocarcinogenesis MOA (Heintz *et al.*, 2023). In our companion article (Heintz *et al.*, 2024), *in vitro* transcriptomic analyses for HFPO-DA support the MOA conclusions from *in vivo* data by demonstrating that transcriptomic responses in HFPO-DA-treated mouse and rat hepatocytes have high concordance with responses in rodent hepatocytes treated with the prototypical PPARα agonist, GW7647. Moreover, there is a lack of concordance of responses in rodent hepatocytes treated with the PPARγ agonist, rosiglitazone, and cytotoxic agents, acetaminophen and d-galactosamine (Heintz *et al.*, 2024). Just as GW7647 served as a positive control for PPARα activation, these latter agents served as positive controls for comparing transcriptomic responses/signatures to assess alternate MOAs involving PPARγ or cytotoxicity that have been hypothesized for HFPO-DA-mediated liver effects in mice (USEPA, 2021).

The established PPARα MOA (currently under development as an adverse outcome pathway) for rodent liver tumors consists of 4 key events: (1) PPARα activation, (2) alteration in cell growth pathways, (3) perturbation of cell growth and survival, and (4) selective clonal expansion of preneoplastic foci cells (Corton *et al.*, 2014, 2018). To further investigate the role of Key Event 1 (PPARα activation) in the MOA for HFPO-DA in mouse liver and elucidate whether HFPO-DA-mediated transcriptomic responses in mouse liver are PPARα-dependent, an additional *in vitro* transcriptomic study was conducted using a similar experimental design as described previously (see companion publication) but with primary PPARα knockout (KO) and wild-type (WT) B6129SF2/J mouse hepatocytes. The PPARα KO mouse is a well-characterized model used to investigate the role of PPARα and molecular mechanisms of putative PPARα activators (Attema *et al.*, 2022; Foreman *et al.*, 2009; Nakagawa *et al.*, 2012; Rosen *et al.*, 2008; Wolf *et al.*, 2008); however, few studies have been conducted in PPARα KO hepatocytes. Upon treatment with known PPARα agonists, PPARα KO mice lack induction of β-oxidation enzyme gene expression, peroxisome proliferation, and hepatomegaly, but increase hepatic lipid accumulation (Lee *et al.*, 1995). Herein, transcriptomic responses in WT and PPARα KO mouse hepatocytes were compared across treatments with HFPO-DA, GW7647, rosiglitazone, acetaminophen, or d-galactosamine following exposure from 12 to 72 h (Figure 1). Results from this study provide novel insight into the mechanisms of PPAR activators and may explain, in part, why some findings in PPARα KO mouse hepatocytes share some similarity to WT counterparts.

**Figure 1.**
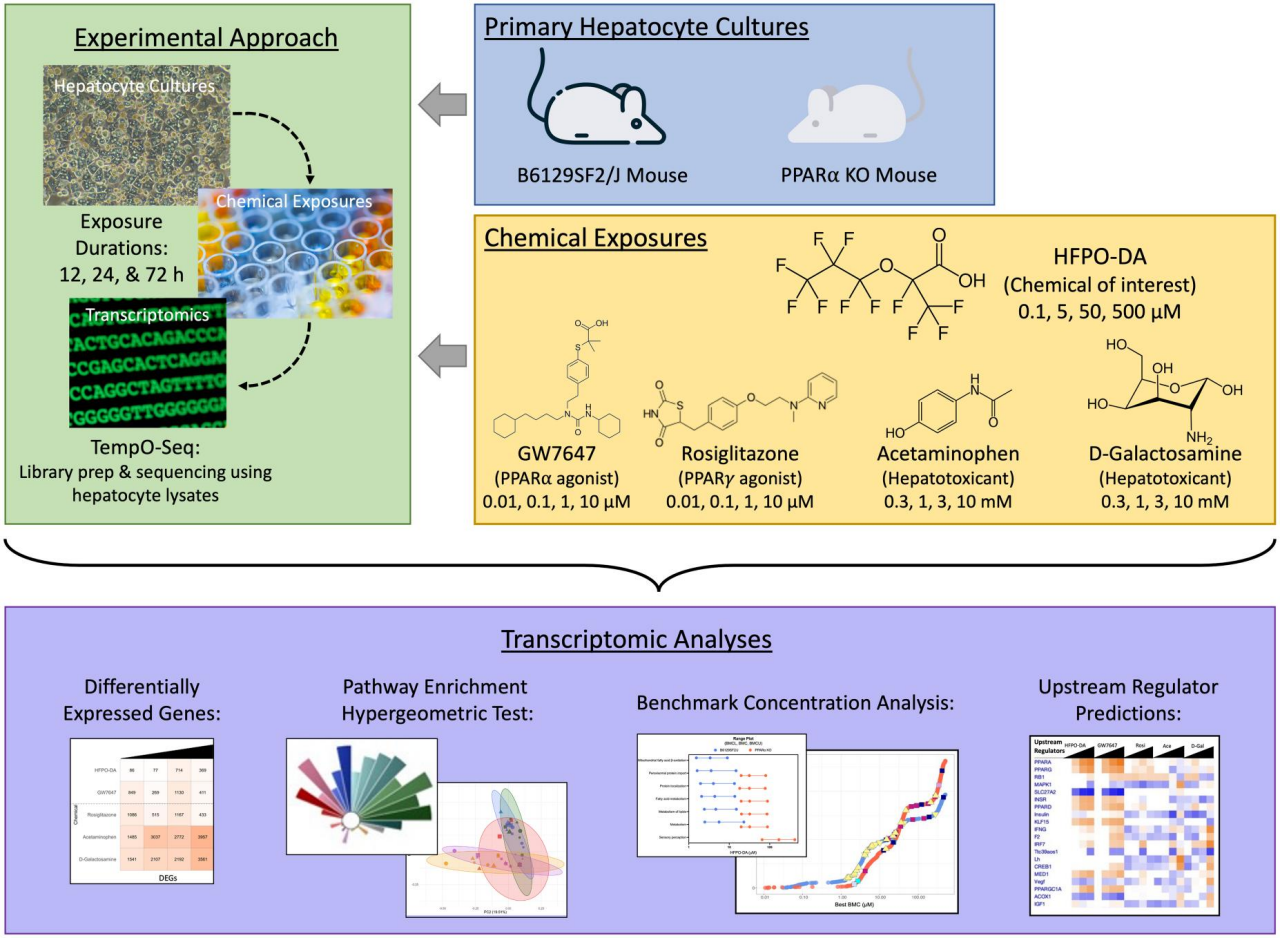
Experimental study design.

## Materials and methods

###  

####  

##### Chemicals

Ammonium perfluoro(2-methyl-3-oxahexanoate) (HFPO-DA; CASRN 62037-80-3; 95% purity) was purchased from Manchester Organics Ltd (Runcorn, Cheshire, United Kingdom). GW7647 (CASRN 265129-71-3; ≥98% purity) was purchased from Cayman Chemical Company (Ann Arbor, Michigan). Rosiglitazone (CASRN 122320-73-4; 98.9% purity), acetaminophen (CASRN 103-90-2; ≥99% purity), and d-galactosamine (CASRN 1772-03-8; ≥99% purity) were purchased from Sigma Aldrich (St Louis, Missouri).

##### Primary hepatocyte isolation and culture

Mouse hepatocytes were isolated from the livers of 11-week-old male B6129SF2/J mice (stock no. 101045) and 11-week-old male PPARα-null mice (B6; 129S4-Ppara^tm1Gonz^/J, stock no. no. 008154) purchased from The Jackson Laboratory (Bar Harbor, Maine). Primary hepatocytes from male mice were used for the *in vitro* assay herein based on findings from previous toxicity studies in rodents demonstrating greater sensitivity to HFPO-DA-mediated liver effects in males (Chappell *et al.*, 2020; Heintz *et al.*, 2022; Thompson *et al.*, 2019). As described in Lee *et al.* (1995), PPARα-null mice were generated by a targeted disruption of the ligand-binding domain (ie, deletion of 83 base pairs in exon 8; see Lee *et al.*, 1995 for details) of the mouse PPARα (mPPARα) gene, rendering the mPPARα gene nonfunctional. Although nonfunctional, mPPARα RNA was detected by Lee *et al.* (1995) in these mice at very low expression levels. However, Lee *et al.* (1995) used Western blotting to confirm the lack of mPPARα protein expression, and lack functional protein activity was demonstrated by the inability to activate downstream PPARα target genes (Lee *et al.*, 1995). PPARα-null mice are considered constitutive KO mice (ie, PPARα is nonfunctional in the entire animal), thus primary hepatocytes from PPARα-null mice will be referred to as PPARα KO hepatocytes. B6129SF2/J mice were used as the genetic background strain for PPARα KO mice^1^ and are referred to as WT hepatocytes.

Hepatocytes from both mouse genotypes were isolated using a 2-step enzymatic digestion of liver tissue as described in Mudra and Parkinson (2001). Hepatocyte viability was determined by trypan blue (0.04%; Millipore Sigma, St Louis, Missouri) exclusion and was ≥79%. Primary hepatocytes were plated in a collagen-sandwich configuration on 48-well plates and maintained according to the methods described in the companion publication.

##### Hepatocyte treatments

Using 48-well plates, WT and PPARα KO mouse hepatocytes were seeded at densities of 0.6 × 10^6^ cells/ml and 0.5 × 10^6^ cells/ml, respectively. After a 24-h acclimatization period, hepatocytes from each genotype were treated for 12, 24, or 72 h with supplemented modified Eagle’s medium containing solvent control in the presence or absence of HFPO-DA (0.1, 5, 50, or 500 μM) or one of the following positive controls: GW7647 (0.01, 0.1, 1, or 10 μM), rosiglitazone (0.01, 0.1, 1, or 10 μM), acetaminophen (0.3, 1, 3, or 10 mM), or d-galactosamine (0.3, 1, 3, or 10 mM). Deionized water (1%) served as the solvent control for HFPO-DA and dimethylsulfoxide (0.1%; cell culture grade; Sigma Aldrich) served as the solvent control for the remaining test chemicals. Treatment solutions were replaced every 24 h. For each genotype, treatment groups were performed in triplicate wells for 12 and 72 h treatment durations, and quadruplicate wells for the 24-h treatment duration.

Hepatocyte cultures were visualized and photographed at 24, 48, and 72 h following treatment to document morphological integrity according to the methods described in the companion publication.

##### Cytotoxicity assay

The release of lactate dehydrogenase (LDH) into culture medium indicates loss of cell membrane integrity and was used to estimate cytotoxicity. LDH release was measured using a commercial kit (Roche Diagnostics GmbH, Mannheim, Germany) according to the manufacturer’s directions. Additional details regarding LDH assay methodology are provided in the companion publication. Cytotoxicity in a treatment group was determined based on measurements of percent LDH release ≥25% in combination with changes in hepatocyte morphology indicative of cytotoxicity. A preliminary cytotoxicity assay was performed to select treatment concentrations used in the present study (data not shown).

##### RNA preparation and sequencing

Following treatment with HFPO-DA or positive controls (ie, GW7647, rosiglitazone, acetaminophen, or d-galactosamine) for 12, 24, or 72 h, hepatocytes were lysed using TempO-Seq Enhanced Lysis Buffer and processed according to the TempO-Seq protocol by BioSypder Technologies (Carlsbad, California), as previously described (Yeakley *et al.*, 2017). Resultant DNA libraries were sequenced using a HiSeq 2500 Ultra-High-Throughput Sequencing System (Illumina, San Diego, California).

##### Sequencing data processing and assessment of quality

Raw sequencing data (ie, FASTQ files) were analyzed according to the TempO-Seq data analysis pipeline described in Yeakley *et al.* (2017). For each mouse genotype, the output from the TempO-Seq pipeline was a table containing the number of sequenced reads per TempO-Seq probe per sample, with each probe representing a gene-specific sequence. Samples were excluded from the downstream analyses if either or both of the following exclusion criteria were met: (1) overall sequencing depth (total number of reads across all probes) lower than 2 SDs below the mean sequencing depth across all samples from the same genotype; (2) total number of sequenced probes lower than 2 SDs below the mean number of probes sequenced per sample from the same genotype. Count data from all samples that were not excluded were used for further comparative analyses.

##### Differential gene expression analyses

Sequencing data were analyzed using packages (described here and in subsequent sections) in the R software environment, version 4.3.1 (cran.r-project.org/). The DESeq2 R package (v1.40.2) (Love *et al.*, 2014) was used to normalize data and account for sample-to-sample variation in sequencing depth within each mouse genotype. Fold-change and differentially expressed probes (DEPs) associated with chemical treatment were determined within DESeq2 by conducting statistical comparisons between treatment groups and controls from the same mouse genotype and treatment duration. DEPs were defined as those with a false discovery rate (FDR) <10%, based on *p*-values adjusted for multiple testing using the Benjamini and Hochberg (BH) procedure (Love *et al.*, 2014); differentially expressed genes (DEGs) were identified from respective DEPs, as some genes (but not all) are represented by multiple probes in the TempO-Seq assay. The expression levels of 21 398 mouse genes as measured by 30 146 mouse probes were reported from the TempO-Seq assay for each sample.

##### Identification of pathway-level responses to treatment

Biological pathways associated with transcriptomic responses in mouse hepatocytes following treatment with HFPO-DA or positive controls were identified by gene set enrichment analysis as described in the companion publication. Enrichment of sets of genes (ie, the constituents of a molecular signaling pathway) was evaluated using the hypergeometric test method for overrepresentation. Significant DEGs (ie, genes with an FDR of <10% as described above) for each treatment group, timepoint, and mouse genotype were tested for overrepresentation among the gene sets in the canonical pathway subcollection using the Fisher combined probability test function within the Platform for Integrative Analysis of Omics data (PIANO) R package (v2.16.0) (Varemo *et al.*, 2013). Gene sets with an FDR <5% were considered significantly enriched.

##### Gene set aggregation and visualizations

A comparative targeted gene set analysis was conducted according to the methods described in the companion publication (Heintz *et al.*, 2024) to better understand the types of gene sets that were significantly enriched across chemical treatment groups. Briefly, the top 10 chemical-gene interactions from the Comparative Toxicogenomics Database (CTD; https://ctdbase.org/; accessed November 2022) for HFPO-DA and each of the positive control chemicals were used to identify and select gene sets that contained one or more of these top interacting genes. From the list of gene sets containing one or more interacting genes, adjusted *p*-values for significantly enriched gene sets (FDR <5%) across all treatment groups were reverse log-scaled, with nonsignificant pathways set equal to zero, and the gene set with the lowest adjusted *p*-value (ie, most significant) set equal to 1. Within each treatment group, gene sets containing the same interacting gene were aggregated by summing the reverse log-scaled adjusted *p*-values. Lastly, the summed totals for each interacting gene for each treatment group and timepoint were scaled from 0 to 1 using internal and external scaling methods. Internal scaling was defined as scaling the summed totals for each interacting gene relative to each other within the same treatment group and timepoint. External scaling was defined as scaling the summed totals for the same interacting gene across all treatment groups and timepoints within a genotype. Once data were appropriately scaled, ToxPi visualizations were generated using ToxPi software (https://toxpi.org/; v2.3).

##### Upstream regulator prediction analyses

Ingenuity Pathway Analysis (IPA, v. 01-22-01; Qiagen Bioinformatics, Redwood City, California) was used to identify predicted upstream regulators associated with DEGs for each treatment group, timepoint, and mouse genotype. Fold change and statistical values determined by DESeq2 were used to conduct the analyses, specifically DEGs with FDR <10%.

##### Benchmark concentration analyses

BMDExpress software (v2.3) (Phillips *et al.*, 2019) was used to perform concentration-response modeling to identify genes altered by chemical treatment in mouse hepatocytes according to the methods described in the companion publication (Heintz *et al.*, 2024). Concentration-responsive genes with a best benchmark concentration (BMC) >10-fold below the lowest concentration or a best BMC>the highest concentration were removed (NTP, 2018). Functional classification of significantly concentration-responsive genes (ie, genes with a winning model fit *p*-value ≥.1) was conducted using the Reactome gene set collections available within the BMDExpress software. Genes were removed according to the default parameters as follows: genes with BMC/BMCL >20, BMCU/BMC >20, and BMCU/BMCL >40 (NTP, 2018). No filters for minimum or maximum number of genes per gene set were applied. BMCs for the gene sets were also calculated.

## Results

###  

#### Transcriptomic changes associated with treatment in WT and PPAR KO mouse hepatocytes

Using the criteria described in the Materials and Methods section for the assessment of sequencing data quality, 7 samples were removed from the analysis for WT mouse hepatocytes and 10 samples were removed for PPARα KO mouse hepatocytes. Final sample numbers for each treatment group included in downstream transcriptomic analyses are provided in Supplementary Table 1 and Supplementary File 1. In general, samples removed across mouse genotypes were from different chemical treatment groups and timepoints; however, at 72 h, all three 10 mM (highest concentration tested) samples for acetaminophen in WT and PPARα KO hepatocytes did not meet sequencing data quality criteria and were removed from the analysis. Findings regarding poor sequencing data quality in the 10-mM acetaminophen treatment group at 72 h for both WT and PPARα KO hepatocytes are consistent with cytotoxicity results (available in Supplementary File 2 and Supplementary Figure 1). In addition, at 12 h, 2 of the 3 total samples from the 1-mM acetaminophen treatment group in PPARα KO hepatocytes did not meet sequencing data quality criteria, as a result, this treatment group was also removed from the analysis. Cytotoxicity was primarily observed in hepatocytes treated with acetaminophen or d-galactosamine, the positive controls for a cytotoxic MOA. As expected, cytotoxicity increased with exposure duration (ie, later timepoints, 24 and 72 h) and concentration (ie, 3 and 10 mM) for acetaminophen and d-galactosamine across hepatocyte genotypes (Supplementary File 2 and Supplementary Figure 1).

The variance in transcriptomic profiles between each sample across chemical treatment groups and timepoints in WT or PPARα KO hepatocytes was visualized using principal component analysis (PCA) (Supplementary Figs. 2–4). PCA results were consistent with findings in the companion publication (Heintz *et al.*, 2024), with the greatest variance attributed to treatment duration (ie, timepoint). Chemical treatment group also contributed to the observed variation between samples, with acetaminophen and d-galactosamine-treated samples having the greatest separation from the other chemical groups in both WT and PPARα KO hepatocytes, particularly at 24 and 72 h timepoints (Supplementary Figure 3). Samples from HFPO-DA, GW7647, and rosiglitazone treatment groups clustered more closely with solvent control samples within each timepoint; however, upon removal of samples treated with acetaminophen and d-galactosamine, HFPO-DA and GW7647-treated samples from WT hepatocytes generally clustered together and separately from rosiglitazone and control samples at 12 and 24 h (Supplementary Figure 4). A distinct clustering pattern following removal of samples treated with acetaminophen and d-galactosamine was not observed in samples from WT hepatocytes at 72 h or in samples from PPARα KO hepatocytes. PCA results were consistent with hierarchical clustering patterns observed within each hepatocyte genotype (Supplementary Figs. 5A and B); in addition, as expected, samples generally grouped by hepatocyte genotype followed by timepoint when all samples were analyzed together by hierarchical clustering (Supplementary Figure 5C).

**Figure 2.**
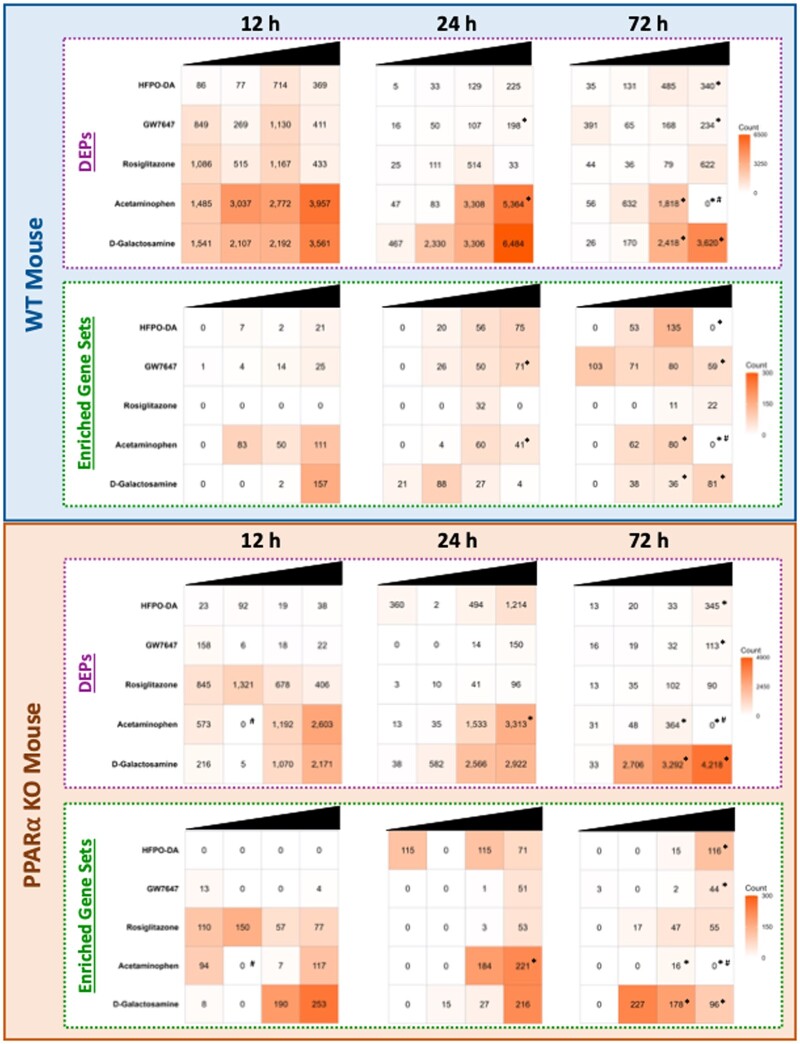
Number of significantly upregulated DEPs (FDR<10%) and enriched gene sets (FDR<5%) for each chemical tested (relative to controls) in WT and PPARα KO mouse hepatocytes across 12, 24, and 72 h. Each row represents a different chemical and each column represents a different test concentration, with concentrations increasing from left to right. An “*” indicates that cytotoxicity was observed at this concentration and timepoint. An “#” indicates that samples from this concentration and timepoint did not undergo transcriptomic analyses due to low sequencing quality.

The number of significantly upregulated DEPs across timepoints for each chemical treatment group in WT and PPARα KO hepatocytes are presented in Figure 2 (results for downregulated DEPs available in Supplementary Figure 6; also available in Supplementary File 3). Consistent with results presented in the companion publication (Heintz *et al.*, 2024), hepatocytes treated with acetaminophen and d-galactosamine generally had the greatest number of DEPs at each timepoint compared with the other chemicals tested.

Across timepoints, the number of DEPs was comparable for HFPO-DA and GW7647, and to a lesser extent rosiglitazone, in WT hepatocytes. In PPARα KO hepatocytes, the number of DEPs was more variable between HFPO-DA, GW7647, and rosiglitazone. At 12 h, rosiglitazone exhibited a high number of DEPs, whereas HFPO-DA and GW7647 had a low number of DEPs. At 24 h, a greater number of DEPs were observed in HFPO-DA-treated PPARα KO hepatocytes than GW7647 or rosiglitazone counterparts; however, by 72 h, the number of DEPs was comparable between all 3 chemicals.

When comparing across genotypes, at 12 h, fewer significantly altered DEPs were observed in both HFPO-DA and GW7647-treated PPARα KO hepatocytes compared with WT counterparts. Conversely, rosiglitazone had a comparable number of DEPs between hepatocyte genotypes at 12 h. At 24 h, there were more DEPs in PPARα KO hepatocytes exposed to HFPO-DA than WT hepatocytes. A comparable number of probes were differentially expressed between genotypes in GW7647 and rosiglitazone-treated hepatocytes at 24 h, and by 72 h, a lower number of DEPs were observed for all 3 chemicals in PPARα KO hepatocytes compared with WT counterparts.

Overall, treatment duration had the greatest impact on variance in transcriptomic profiles between hepatocyte samples for both WT and PPARα KO hepatocytes. Mouse hepatocytes treated with acetaminophen or d-galactosamine generally had a different and greater transcriptomic response based on PCA and total differential gene expression counts compared with the other chemicals tested. In addition, a difference in the number of DEPs between genotypes was observed at the early timepoints for HFPO-DA and GW7647-treated hepatocytes.

#### Comparison of pathway-level responses to HFPO-DA and positive controls in WT and PPARα KO mouse hepatocytes

The number of significant (FDR<5%) upregulated gene sets differed between WT and PPARα KO hepatocytes that received the same treatment, particularly at the 12-h timepoint for HFPO-DA, GW7647, and rosiglitazone-treated hepatocytes (Figure 2, Tables 1–3; Supplementary File 4). In WT hepatocytes, the number of significantly upregulated gene sets increased with increasing concentration for both HFPO-DA and GW7647 across timepoints, apart from 72 h, where changes in hepatocyte morphology and LDH release, indicative of cell death, were observed at the highest concentrations. Enriched gene sets were similar between the 2 chemicals across timepoints and related to β-oxidation of fatty acids and PPAR signaling (see Tables 1–3). Conversely, in PPARα KO hepatocytes at 12 h, zero gene sets were upregulated following HFPO-DA treatment, and only a few gene sets were upregulated in the lowest and highest concentration groups for GW7647. The 13 enriched gene sets in the lowest concentration group for GW7647 (0.01 µM) were specifically related to mitochondrial stress, whereas the 4 gene sets upregulated in the highest concentration group (10 µM) were related to mitochondrial fatty acid β-oxidation. Responses observed in the lowest concentration group for GW7647 at 12 h may be indicative of a mitohormetic response, because responses at higher concentrations and later timepoints did not include a mitochondrial stress response and were instead related to PPARα signaling and mitochondrial β-oxidation (Cox *et al.*, 2018). Regardless of the types of enriched gene sets, the adjusted *p*-values for all enriched gene sets in GW7647-treated PPARα KO hepatocytes at 12 h were at least 6-fold greater (ie, 6-fold lower significance) than adjusted *p*-values for upregulated gene sets in WT counterparts, indicating a lower transcriptomic response (Table 1).

**Table 1.** Top 5 most significant upregulated gene sets across treatment concentrations at 12 h in WT and PPARα KO mouse hepatocytes

	WT			PPARα KO		
Chemical	Concentration	Upregulated gene set	Adjusted *p*-value	Concentration	Upregulated gene set	Adjusted *p*-value
HFPO-DA	500 µM	KEGG fatty acid metabolism	8.38E-10	*NS*	*NS*	*NS*
	500 µM	WP fatty acid β-oxidation	1.72E-08			
	500 µM	WP mitochondrial long-chain fatty acid β-oxidation	2.28E-07			
	500 µM	REACTOME fatty acid metabolism	3.92E-07			
	500 µM	KEGG PPAR Signaling Pathway	2.13E-06			
GW7647	10 µM	WP fatty acid β-oxidation	7.83E-11	0.01 µM	KEGG Alzheimer’s Disease	.0002597
	10 µM	REACTOME fatty acid metabolism	8.26E-11	0.01 µM	KEGG Oxidative Phosphorylation	.001082
	10 µM	WP PPAR signaling pathway	1.92E-09	0.01 µM	REACTOME Respiratory Electron Transport ATP Synthesis by Chemiosmotic Coupling and Heat Production by Uncoupling Proteins	.001082
	10 µM	KEGG PPAR signaling pathway	2.28E-09	0.01 µM	WP Electron Transport Chain Oxidative Phosphorylation System in Mitochondria	.001238
	10 µM	WP mitochondrial long-chain fatty acid β-oxidation	4.30E-09	10 µM	WP Mitochondrial Long-Chain Fatty Acid β-oxidation	.004178
Rosiglitazone	*NS*	*NS*	*NS*	0.1 µM	REACTOME Translation	1.61E-15
				0.01 µM	REACTOME Regulation of Expression of SLITS and ROBOS	7.00E-11
				0.01 µM	REACTOME Translation	7.00E-11
				0.1 µM	REACTOME Regulation of Expression of SLITS and ROBOS	1.63E-09
				0.01 µM	REACTOME Signaling of ROBO Receptors	1.91E-09
Acetaminophen	10 mM	REACTOME cellular response to starvation	2.76E-08	10 mM	REACTOME Cellular Response to Starvation	2.95E-27
	10 mM	REACTOME influenza infection	1.39E-07	10 mM	REACTOME Response of EIF2AK4 (GCN2) to Amino Acid Deficiency	2.95E-27
	10 mM	REACTOME response of EIF2AK4 (GCN2) to amino acid deficiency	2.16E-07	10 mM	REACTOME Eukaryotic Translation Initiation	1.18E-26
	10 mM	REACTOME regulation of IFNA/IFNB signaling	1.74E-06	10 mM	REACTOME Eukaryotic Translation Elongation	4.01E-25
	1 mM	REACTOME nuclear events mediated by NFE2L2	3.20E-06	10 mM	REACTOME Nonsense Mediated Decay	2.42E-23
D-Galactosamine	10 mM	REACTOME metabolism of amino acids and derivatives	1.64E-12	10 mM	REACTOME Translation	1.12E-15
	10 mM	REACTOME translation	1.98E-12	10 mM	REACTOME Eukaryotic Translation Initiation	4.45E-14
	10 mM	REACTOME regulation of expression of SLITS and ROBOS	1.51E-10	10 mM	REACTOME Metabolism of Amino Acids and Derivatives	1.05E-13
	10 mM	REACTOME eukaryotic translation initiation	8.50E-10	10 mM	WP VEGF-A/VEGFR-2 Signaling Pathway	3.32E-12
	10 mM	REACTOME eukaryotic translation elongation	1.09E-09	10 mM	REACTOME Selenoamino Acid Metabolism	4.02E-11

Abbreviations: *NS*, not significant, ie, gene sets did not meet significance threshold (adjusted *p*-value<.05) for gene set enrichment.

**Table 2.** Top 5 most significant upregulated gene sets across treatment concentrations at 24 h in WT and PPARα KO mouse hepatocytes

	WT			PPARα KO		
Chemical	Concentration	Upregulated gene set	Adjusted *p*-value	Concentration	Upregulated gene set	Adjusted *p*-value
HFPO-DA	500 µM	KEGG fatty acid metabolism	1.10E-26	500µM	KEGG Fatty Acid Metabolism	5.79E-10
	50 µM	REACTOME fatty acid metabolism	4.36E-22	50µM	KEGG Fatty Acid Metabolism	8.74E-08
	500 µM	REACTOME fatty acid metabolism	4.82E-22	50µM	REACTOME Fatty Acid Metabolism	3.57E-07
	500 µM	KEGG fatty acid metabolism	5.83E-20	50µM	REACTOME Metabolism of Amino Acids and Derivatives	9.67E-07
	500 µM	KEGG PPAR signaling pathways	2.50E-15	0.1 µM	REACTOME ABC Transporter Disorders	4.57E-06
GW7647	10 µM	KEGG fatty acid metabolism	7.55E-30	10 µM	REACTOME Fatty Acid Metabolism	5.44E-20
	10 µM	REACTOME fatty acid metabolism	1.50E-23	10 µM	KEGG PPAR Signaling Pathways	3.55E-14
	10 µM	KEGG PPAR signaling pathway	5.40E-21	10 µM	WP PPAR Signaling Pathways	5.27E-13
	10 µM	WP PPAR signaling pathway	9.31E-20	10 µM	KEGG Fatty Acid Metabolism	4.05E-11
	1 µM	KEGG fatty acid metabolism	6.72E-15	10 µM	KEGG Peroxisome	2.04E-09
Rosiglitazone	1 µM	REACTOME regulation of IFNA/B signaling	8.02E-15	10 µM	REACTOME Fatty Acid Metabolism	8.78E-18
	1 µM	WP SARS coronavirus and innate immunity	1.40E-13	10 µM	KEGG PPAR Signaling Pathway	7.30E-15
	1 µM	REACTOME TRAF6 mediated IRF7 activation	5.24E-11	10 µM	WP_PPAR Signaling Pathway	1.98E-13
	1 µM	WP overview of interferons mediated signaling pathway	5.24E-11	10 µM	REACTOME Peroxisomal Lipid Metabolism	4.63E-10
	1 µM	REACTOME interferon alpha/beta signaling	5.35E-11	10 µM	REACTOME Peroxisomal Protein Import	6.22E-09
Acetaminophen	10 mM	REACTOME cellular response to starvation	4.50E-07	10 mM	REACTOME Regulation of Expression of SLITs and ROBOs	2.47E-27
	10 mM	REACTOME response of EIF2AK4 (GCN2) to amino acid deficiency	4.50E-07	10 mM	REACTOME Nonsense Mediated Decay (NMD)	1.51E-24
	10 mM	REACTOME eukaryotic translation elongation	8.06E-07	10 mM	REACTOME Influenza Infection	8.56E-24
	10 mM	REACTOME nonsense mediated decay (NMD)	8.51E-07	10 mM	REACTOME Cellular Response to Starvation	3.18E-23
	10 mM	REACTOME eukaryotic translation initiation	1.17E-06	10 mM	REACTOME Eukaryotic Translation Elongation	3.18E-23
D-Galactosamine	0.3 mM	REACTOME metabolism of amino acid and derivatives	2.82E-07	10 mM	REACTOME Translation	4.77E-45
	1 mM	REACTOME eukaryotic translation initiation	1.23E-05	10 mM	REACTOME Eukaryotic Translation Initiation	7.64E-31
	3 mM	REACTOME TRAF6 mediated IRF7 activation	1.26E-05	10 mM	REACTOME Eukaryotic Translation Elongation	2.69E-27
	1 mM	REACTOME regulation of expression of SLITs and ROBOs	2.02E-05	10 mM	REACTOME Regulation of Expression of SLITs and ROBOs	5.40E-26
	0.3 mM	KEGG ribosome	2.09E-05	10 mM	KEGG Ribosome	1.28E-25

**Table 3.** Top 5 most significant upregulated gene sets across treatment concentrations at 72 h in WT and PPARα KO mouse hepatocytes

	WT			PPARα KO		
Chemical	Concentration	Upregulated gene set	Adjusted *p*-value	Concentration	Upregulated gene set	Adjusted *p*-value
HFPO-DA	50 µM	KEGG fatty acid metabolism	4.18E-23	500 µM	REACTOME Fatty Acid Metabolism	4.38E-17
	500µM	REACTOME fatty acid metabolism	4.55E-22	500 µM	KEGG Fatty Acid Metabolism	5.00E-15
	5 µM	KEGG fatty acid metabolism	5.22E-21	500 µM	REACTOME Phase I Functionalization of Compounds	1.35E-10
	5 µM	REACTOME fatty acid metabolism	7.48E-17	500 µM	KEGG PPAR Signaling Pathway	2.80E-10
	5 µM	KEGG PPAR signaling pathway	5.59E-16	500 µM	REACTOME Biological Oxidations	2.80E-10
GW7647	10 µM	KEGG fatty acid metabolism	4.85E-27	10 µM	KEGG PPAR Signaling Pathway	2.73E-16
	1 µM	KEGG fatty acid metabolism	8.01E-26	10 µM	WP PPAR Signaling Pathway	4.57E-15
	0.01 µM	REACTOME fatty acid metabolism	2.02E-25	10 µM	REACTOME Fatty Acid Metabolism	2.33E-14
	1 µM	REACTOM fatty acid metabolism	8.78E-24	10 µM	KEGG Fatty Acid Metabolism	4.61E-11
	0.01 µM	KEGG fatty acid metabolism	7.85E-23	10 µM	WP Fatty Acid Transporters	5.01E-09
Rosiglitazone	1 µM	KEGG PPAR signaling pathway	6.65E-05	10 µM	KEGG PPAR Signaling Pathway	3.89E-16
	1 µM	WP PPARα pathway	6.65E-05	10 µM	REACTOME Fatty Acid Metabolism	3.89E-16
	1 µM	WP PPAR signaling pathway	6.65E-05	10 µM	WP PPAR Signaling Pathway	6.17E-15
	10 µM	KEGG peroxisome	9.63E-05	1 µM	REACTOME Fatty Acid Metabolism	2.10E-14
	10 µM	REACTOME protein localization	1.63E-04	1 µM	KEGG PPAR Signaling Pathway	6.59E-12
Acetaminophen	3 mM	REACTOME metabolism of amino acids and derivatives	2.30E-10	3 mM	WP NRF2 Pathway	2.60E-04
	1 mM	REACTOME metabolism of amino acids and derivatives	2.96E-10	3 mM	REACTOME HSF1 Dependent Transactivation	5.04E-04
	3 mM	WP metabolic reprogramming in colon cancer	1.48E-09	3 mM	WP Ferroptosis	5.04E-04
	1 mM	WP amino acid metabolism	1.36E-08	3 mM	REACTOME Attenuation Phase	5.70E-04
	1 mM	KEGG drug metabolism cytochrome P450	1.00E-07	3 mM	REACTOME HSF1 Activation	9.64E-04
D-Galactosamine	1 mM	WP NRF2 pathway	2.22E-11	1 mM	REACTOME rRNA Processing	3.93E-45
	3 mM	REACTOME rRNA processing	3.34E-10	3 mM	REACTOME rRNA Processing	6.01E-30
	10 mM	REACTOME rRNA processing	1.48E-08	10 mM	REACTOME rRNA Processing	1.53E-27
	3 mM	REACTOME rRNA modification in the nucleus and cytosol	1.98E-08	10 mM	REACTOME Eukaryotic Translation Elongation	2.95E-26
	3 mM	REACTOME chromatin modifying enzymes	2.20E-08	10 mM	REACTOME Eukaryotic Translation Initiation	2.95E-26

At later timepoints (ie, 24 and 72 h), significantly upregulated gene sets for HFPO-DA and GW7647-treated PPARα KO hepatocytes were generally limited to the 2 highest concentration groups. The types of gene sets significantly upregulated by both chemicals in PPARα KO hepatocytes were similar to WT hepatocytes treated with HFPO-DA or GW7647, and were related to PPAR signaling and fatty acid metabolism/β-oxidation (Tables 2 and 3). However, in the lowest concentration group for HFPO-DA-treated PPARα KO hepatocytes at 24 h, genes underlying significantly upregulated gene sets were largely related to protein ubiquitination (eg, *Psma, Psmc, Psmd, Psme*) (Table 2; Supplementary File 4). This response was not observed at higher concentrations or at 72 h.

The apparent initial temporal delay in transcriptomic response to HFPO-DA and GW7647 treatment in PPARα KO hepatocytes at 12 h was not observed in PPARα KO hepatocytes treated with rosiglitazone, acetaminophen, or d-galactosamine. In contrast to the PPARα activators, the PPARγ agonist rosiglitazone increased pathway enrichment across treatment groups at 12 h in PPARα KO hepatocytes but not in WT hepatocytes (Figure 2, Table 1). At higher concentrations and later timepoints, the types of upregulated gene sets in rosiglitazone-treated hepatocytes, especially PPARα KO, were similar to enriched gene sets in HFPO-DA and GW7647-treated hepatocytes and related to PPAR signaling and fatty acid metabolism (see Tables 2 and 3). However, the genes underlying these gene sets are regulated by multiple nuclear receptors involved in maintaining energy homeostasis. For example, the “KEGG PPAR Signaling Pathway” consists of 3 PPAR isoforms (α/δ/γ) and while specific genes are regulated by specific receptors, there is also substantial overlap in regulation, with several genes regulated by all 3 PPAR isoforms (Chappell *et al.*, 2020; Heintz *et al.*, 2022).

In general, the number of significantly enriched gene sets in acetaminophen and d-galactosamine treatment groups was greater in PPARα KO hepatocytes across all timepoints, however, the types of gene sets enriched for each chemical were consistent between mouse genotypes and appeared to be unrelated to fatty acid metabolism or energy homeostasis (Tables 1–3; Supplementary File 4). Fewer gene sets were significantly downregulated (compared with the number upregulated) across timepoints and chemical treatment groups in both hepatocyte genotypes (Supplementary Figure 6).

**Figure 3.**
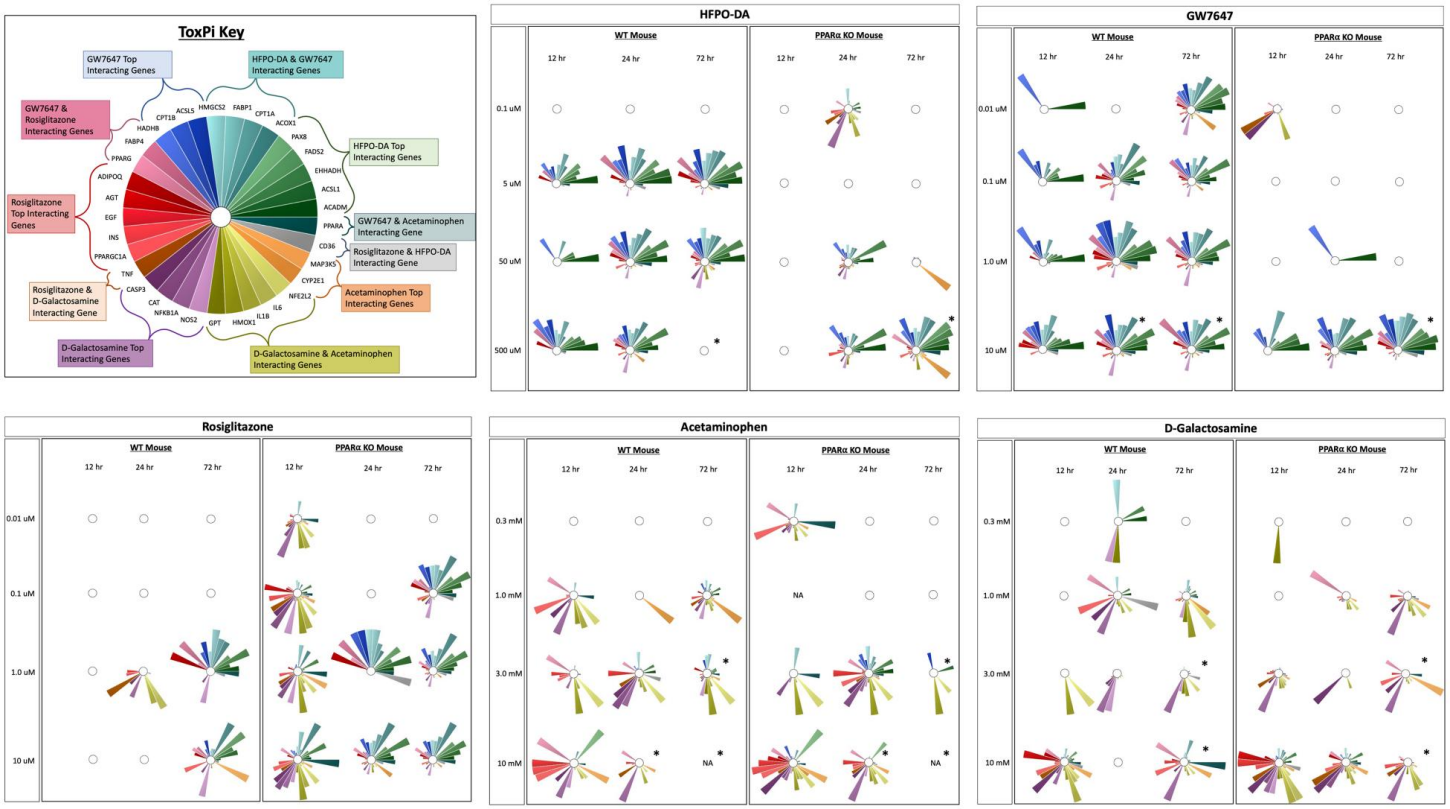
ToxPi visualizations of upregulated gene set aggregation results for WT and PPARα KO mouse hepatocytes using internal scaling approach. Significant (FDR < 5%) upregulated gene sets from hypergeometric gene set enrichment analysis containing genes known to interact with HFPO-DA and/or positive controls were aggregated as described in the Materials and Methods section. The size of a ToxPi wedge for a given gene reflects the significance and number of enriched gene sets containing that gene within a specific chemical treatment group and timepoint that is scaled in respect to the other wedges for different genes within the same ToxPi (ie, internal scaling). An “*” indicates that cytotoxicity was observed at this concentration and timepoint. An empty ToxPi indicates that none of the targeted gene sets were enriched significantly, and “NA” means that samples from this concentration and timepoint did not undergo transcriptomic analyses due to low sequencing quality.

**Figure 4.**
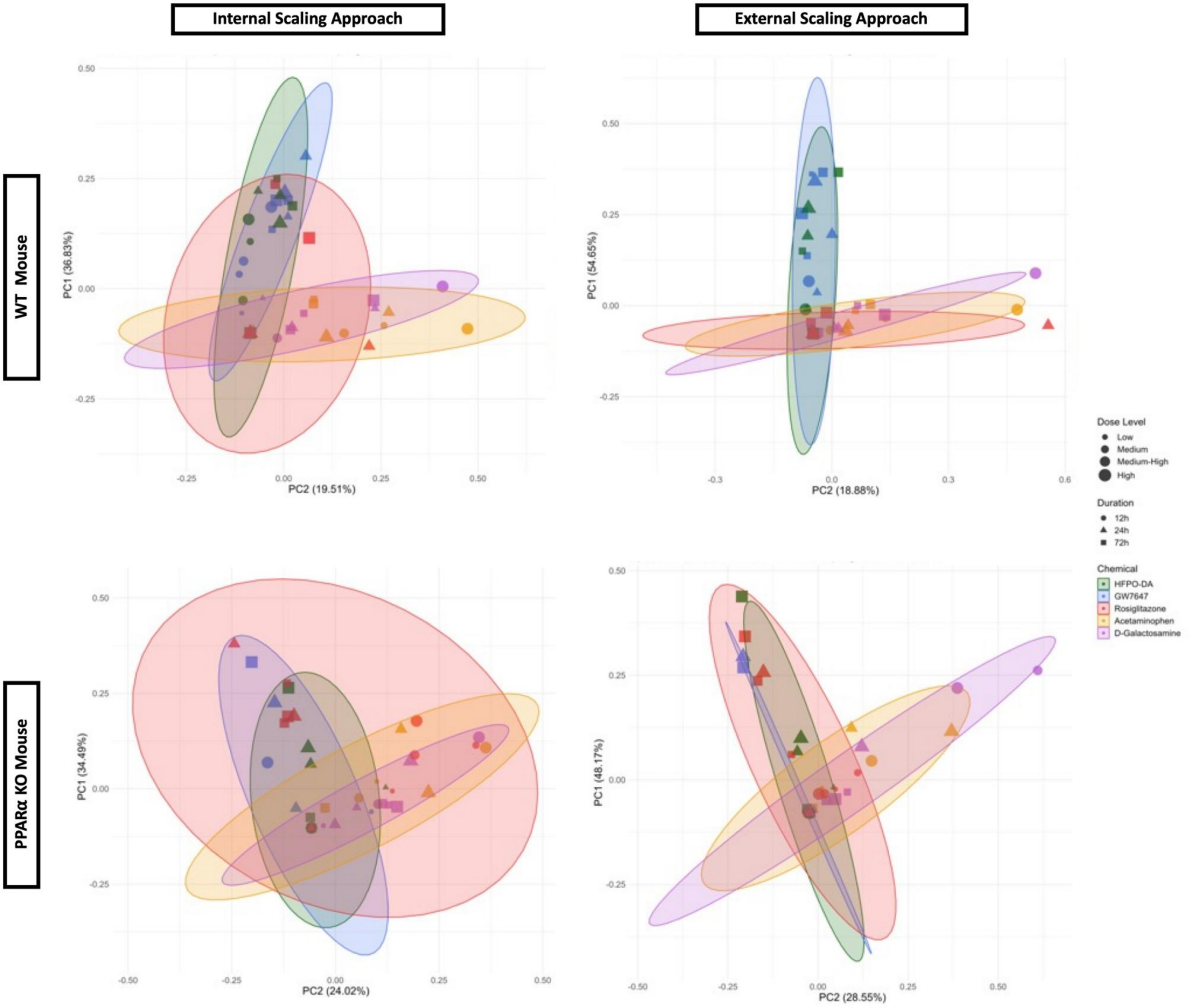
PCA of upregulated gene set aggregation results for WT and PPARα KO mouse hepatocytes using internal and external scaling methods. Shaded ellipses are based on the covariance matrix of the aggregated gene set results with ellipse size accounting for aspects of the underlying cumulative probability distribution, ie, the ellipses provide general insight into the directionality and variance of the respective chemical treatment groups.

Concordance of transcriptomic responses at the pathway level between HFPO-DA and other positive controls was assessed in WT and PPARα KO hepatocytes by aggregating gene sets according to known chemical–gene interactions. Gene set aggregation visuals (ie, ToxPi visuals, PCA plots) for WT hepatocytes were consistent with results observed in hepatocytes from other WT rodent species and strains (see companion publication; Heintz *et al.*, 2024), and demonstrated homogeneous upregulated gene set enrichment profiles for HFPO-DA and GW7647 (ie, similar ToxPi wedge pattern and size across treatment concentrations and timepoints, and greater overlap in PCA plots) using both internal (within a specific chemical treatment group, timepoint, and genotype) and external (across chemical treatment groups and timepoints within a specific genotype) scaling approaches (described in the Materials and Methods section) (Figs. 3 and 4; Supplementary Figure 7). HFPO-DA also had the greatest concordance with GW7647 in PPARα KO hepatocytes by both internal and external scaling approaches.

At higher concentrations and later timepoints, upregulated gene set enrichment profiles for rosiglitazone were more similar to HFPO-DA and GW7647 in PPARα KO hepatocytes, especially when the external scaling approach was used (Figs. 3 and 4). For both hepatocyte genotypes, upregulated gene set enrichment profiles for acetaminophen and d-galactosamine-treated hepatocytes were most concordant with each other (Figure 4); in addition, each cytotoxic agent demonstrated similar ToxPi profile patterns between WT and PPARα KO hepatocytes (Figure 3; Supplementary Figure 7). Due to the lower number of significant downregulated gene sets in WT and PPARα KO hepatocytes across chemical treatment groups and timepoints (Supplementary Figure 6), aggregation analyses of downregulated gene sets were not able to be performed.

Overall, compared with WT hepatocytes, initial transcriptomic responses at the pathway-level were delayed in PPARα KO hepatocytes treated with HFPO-DA or GW7647, as little to no significant gene set enrichment was observed at 12 h for either chemical. This initial temporal delay in transcriptomic response was not observed in rosiglitazone, acetaminophen, or d-galactosamine-treated PPARα KO hepatocytes. At later time points and higher concentrations, ToxPi profiles demonstrated greatest pathway level concordance between HFPO-DA and GW7647-treated hepatocytes in both WT and PPARα KO hepatocytes. In addition, rosiglitazone treatment in PPARα KO hepatocytes resulted in more similar ToxPi profiles to that of HFPO-DA and GW7647 treatment, especially at 24 and 72 h.

#### Comparison of upstream regulator predictions across chemical treatment groups in WT and PPARα KO mouse hepatocytes

**Figure 5.**
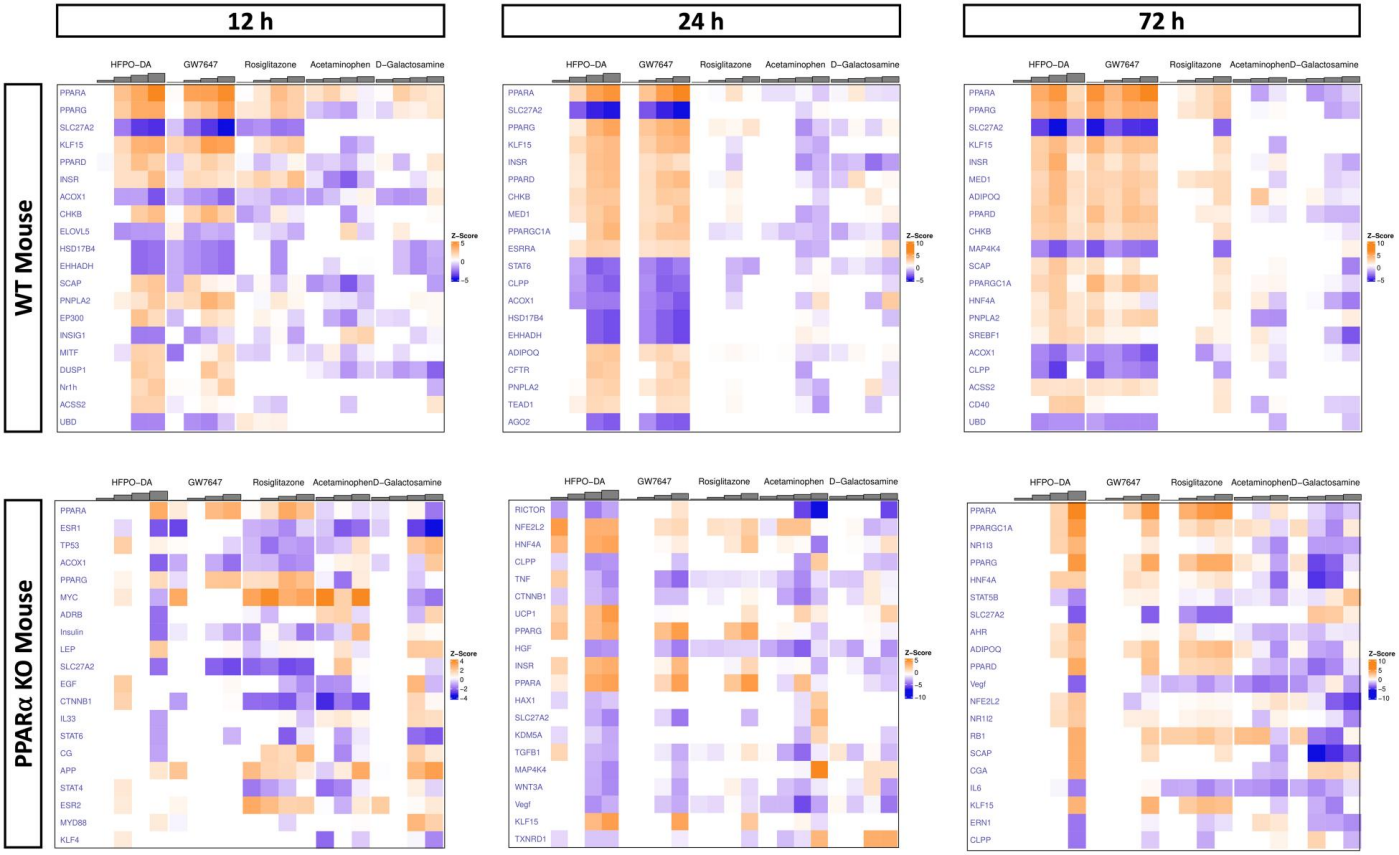
Comparison of activation/inhibition patterns for HFPO-DA’s top 20 predicted upstream regulators across chemical treatment groups at 12, 24, and 72 h. Each column represents a different test concentration, with concentrations increasing from left to right for each chemical. Orange indicates predicted activation, and blue indicates predicted inhibition; the intensity of each color increases with the absolute *z*-score. Columns with no *z*-score prediction indicate chemical treatment groups with a low number DEGs and upstream regulator predictions were not able to be estimated.

The top 20 predicted upstream regulators for HFPO-DA-treated WT and PPARα KO hepatocytes were identified using QIAGEN Ingenuity Pathway Analysis (IPA) for each timepoint. The activation/inhibition *z*-scores for each of these 20 predicted upstream regulators were then compared across chemical treatment groups for each timepoint and hepatocyte genotype (Figure 5). Similar activation/inhibition patterns between HFPO-DA and GW7647 were observed across all 3 timepoints in WT hepatocytes. At 12 h, rosiglitazone-treated WT hepatocytes shared a somewhat consistent predicted upstream regulator pattern to HFPO-DA and GW7647 counterparts. However, by 24 and 72 h, predicted regulator patterns for rosiglitazone were consistent, albeit substantially weaker, than predicted regulator patterns for HFPO-DA and GW7647 in WT hepatocytes. In contrast, patterns of predicted upstream regulator activation/inhibition for acetaminophen and d-galactosamine were generally inconsistent with predicted regulator activation/inhibition patterns for HFPO-DA in WT hepatocytes.

Due to the limited transcriptomic response at earlier timepoints (see Figs. 2 and 3), upstream regulator predictions were reduced for HFPO-DA and GW7647-treated PPARα KO hepatocytes, especially at 12 h (Figure 5). Consistency in upstream regulator prediction patterns between HFPO-DA, GW7647, and rosiglitazone increased across time in PPARα KO hepatocytes. In addition, similar to what was observed in WT hepatocytes, predicted regulator activation/inhibition patterns for acetaminophen and d-galactosamine-treated PPARα KO hepatocytes were inconsistent with patterns observed in HFPO-DA counterparts.

PPARα was the top predicted upstream regulator in WT hepatocytes treated with HFPO-DA or GW7647 across all 3 timepoints (Figure 5; Supplementary Figs. 8–10). PPARα was also the top predicted upstream regulator in PPARα KO hepatocytes at 12 and 72 h for these 2 chemicals (Supplementary Figs. 8 and 10). In addition, PPARα was also predicted as the top regulator for the prototypical PPARγ agonist rosiglitazone at 72 h in PPARα KO hepatocytes (Supplementary Figure 10), which is consistent with gene set enrichment analysis results shown in Table 3.

Overall, upstream regulator prediction patterns for HFPO-DA are most similar to GW7647 in both hepatocyte genotypes, however, at earlier timepoints, regulator predictions for HFPO-DA and GW7647-treated PPARα KO hepatocytes were limited due to a lower number of DEPs. At 72 h, regulator prediction patterns for HFPO-DA and GW7647 were also similar to regulator prediction patterns for rosiglitazone.

#### Benchmark concentration modeling of gene expression data from WT and PPARα KO mouse hepatocytes

**Figure 6.**
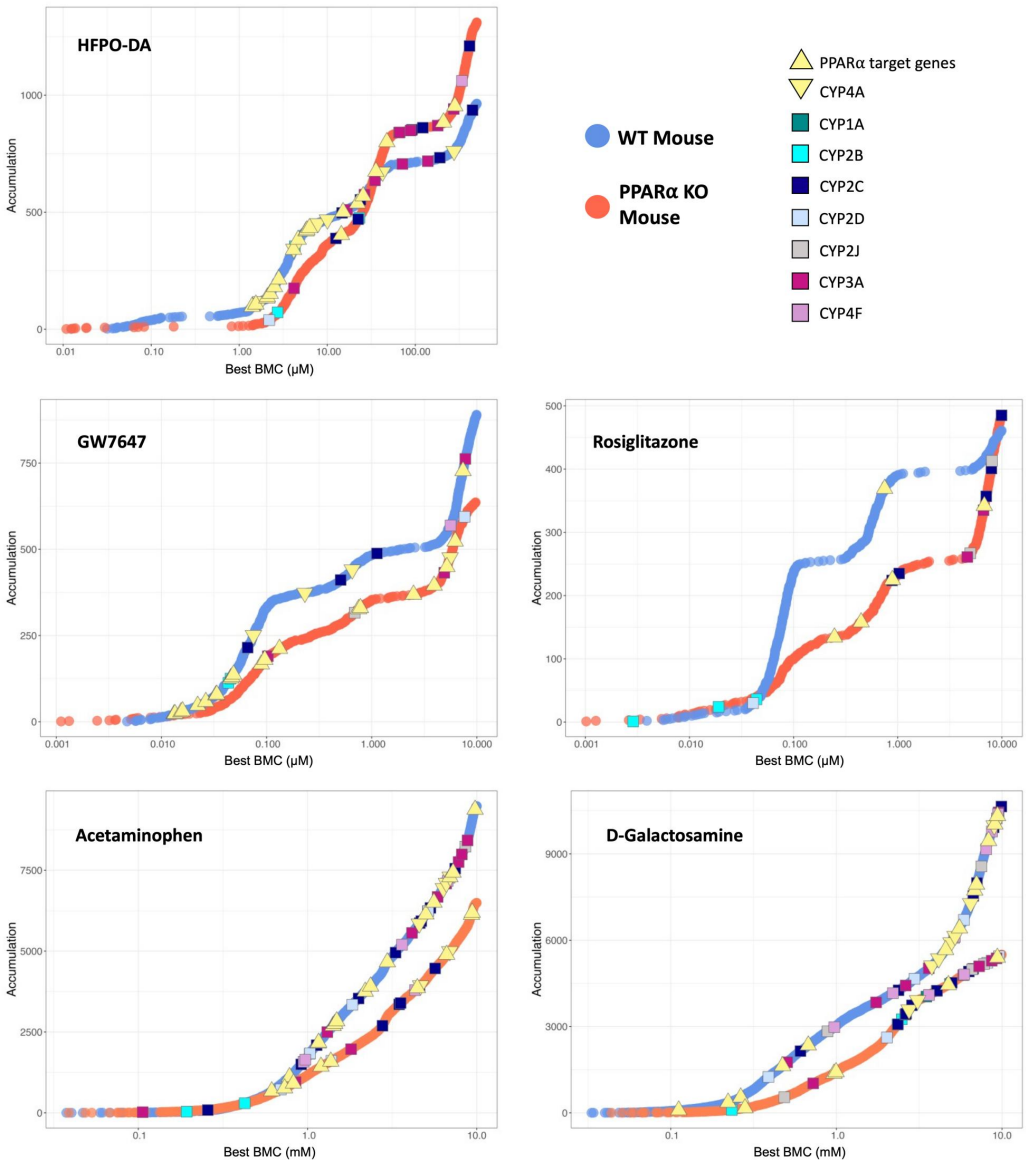
Accumulation plots of best BMCs among significant concentration-responsive probes (best fit *p*-value ≥.1) in WT and PPARα KO mouse hepatocytes at 24 h. Concentration-responsive probes are indicated by blue and orange points for WT and PPARα KO hepatocytes, respectively. Best BMCs of PPARα target genes and lipid metabolizing cytochrome P450’s (CYPs) are annotated by color-coded shapes. PPARα target genes consist of 12 genes identified as HFPO-DA and/or GW7647-interacting genes highlighted in the ToxPi key in Figure 3. CYP4A is set apart from the other lipid metabolizing CYPs (denoted by a yellow downward-facing triangle shape) because it is the target CYP for PPARα.

**Figure 7.**
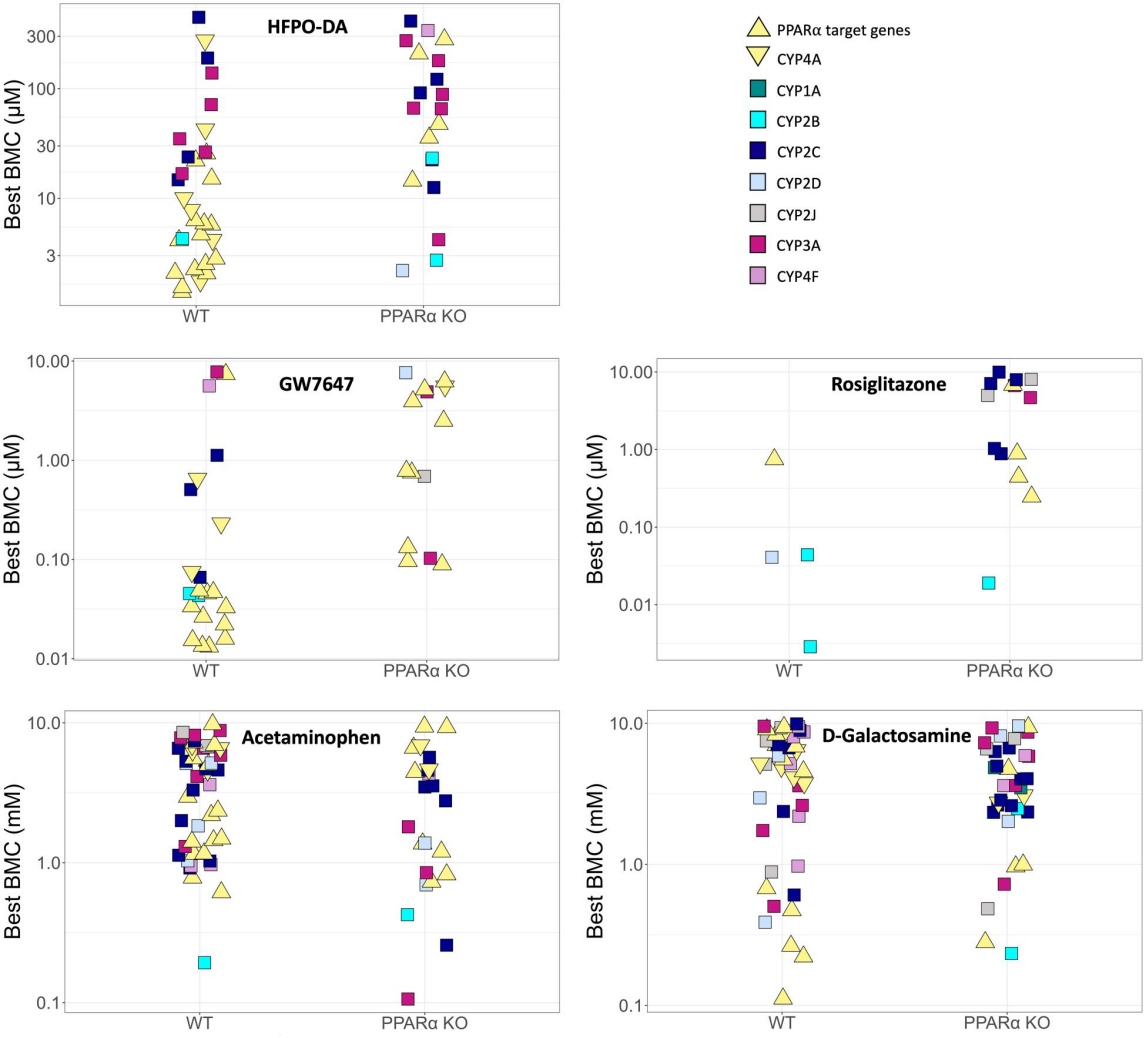
Best BMCs (best fit *p*-value ≥.1) for PPARα target genes and lipid metabolizing CYPs in WT and PPARα KO mouse hepatocytes at 24 h. Best BMCs are annotated by color-coded shapes. PPARα target genes consist of 12 genes identified as HFPO-DA and/or GW7647-interacting genes highlighted in the ToxPi key in Figure 3. CYP4A is set apart from the other lipid metabolizing CYPs (denoted by a yellow downward-facing triangle shape) because it is the target CYP for PPARα.

The concentration-response across all genes for each treatment group and timepoint in WT and PPARα KO hepatocytes was modeled using BMDExpress v2.3 (Phillips *et al.*, 2019) (Supplementary File 5). As shown in Figure 6, there were comparable patterns in BMC values for concentration-response genes in WT and PPARα KO hepatocytes for each of the chemical treatments except for rosiglitazone, where treated WT hepatocytes had a greater number of genes with a best BMC between 0.1 and 10 µM than PPARα KO counterparts. Also highlighted in Figure 6 are the BMC values for various PPARα responsive genes and CYPs involved in lipid metabolism across hepatocyte genotypes and chemical treatment groups at 24 h (results for 12 and 72 h shown in Supplementary Figs. 11 and 12). The BMC values for PPARα target genes were consistently lower than the BMC values for lipid metabolizing CYPs in WT hepatocytes treated with HFPO-DA or GW7647. This was also observed in WT mouse, rat, and human hepatocytes treated with either HFPO-DA or GW7647 (see companion publication). In contrast, the BMC values for PPARα target genes in PPARα KO hepatocytes treated with HFPO-DA or GW7647 were consistently higher (ie, approximately 10-fold greater) than the BMC values in WT counterparts across timepoints (Figs. 6 and 7; Supplementary Figs. 11–13). For example, at 24 h, the lowest BMCs for PPARα target genes in WT hepatocytes were approximately 1.3 and approximately 0.013 µM, for HFPO-DA and GW7647, respectively; whereas, in PPARα KO hepatocytes, the lowest BMCs for PPARα target genes were approximately 14 and approximately 0.1 µM, for HFPO-DA and GW7647, respectively (Figure 7). These results indicate a shift in the activation concentration of PPARα target genes in PPARα KO hepatocytes treated with HFPO-DA or GW7647. This shift in the activation of PPARα target genes was not observed in PPARα KO hepatocytes treated with rosiglitazone, acetaminophen, or d-galactosamine at any timepoint.

**Figure 8.**
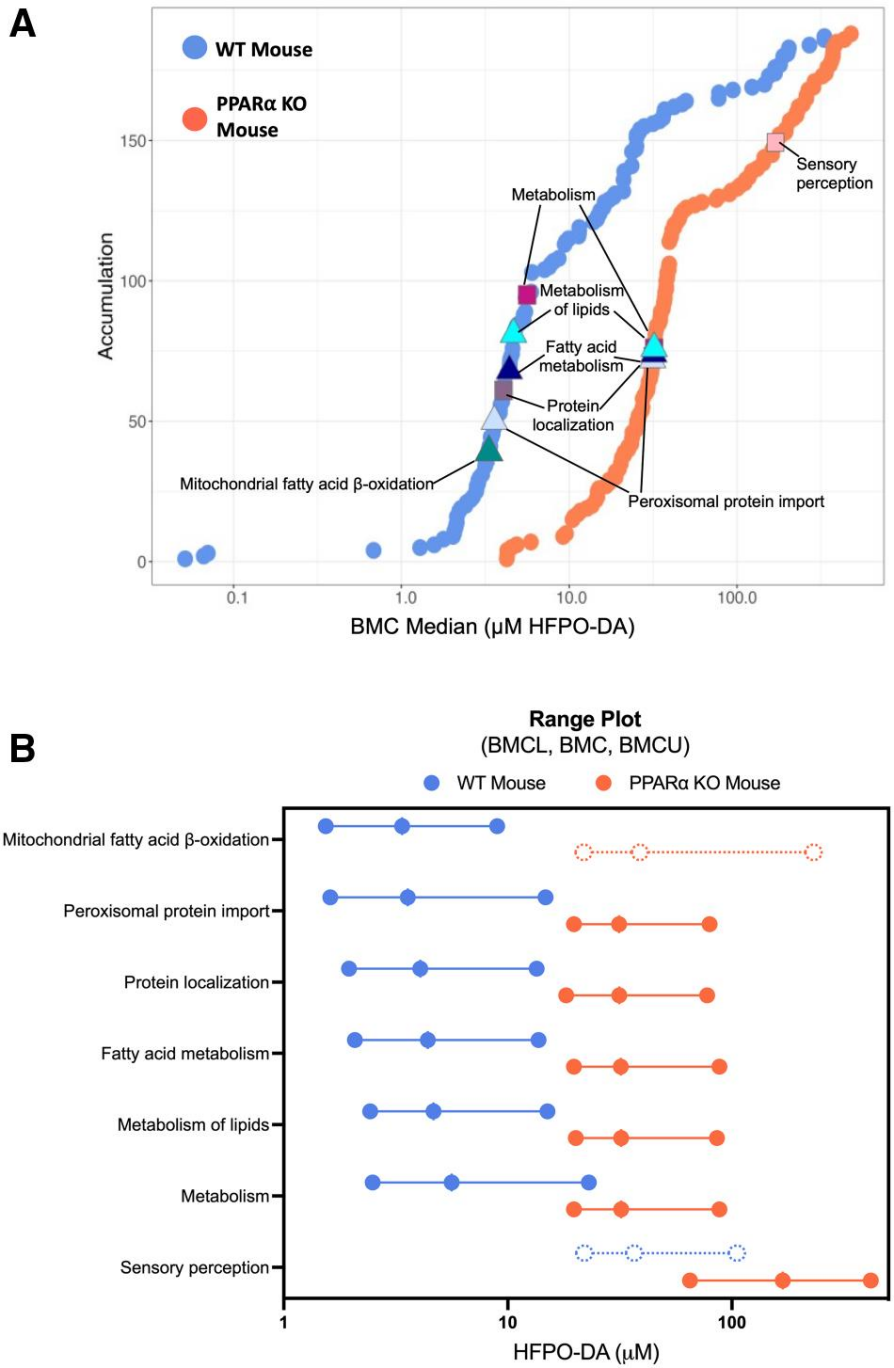
Pathway enrichment of significant concentration-responsive genes in HFPO-DA-treated WT (blue points) and PPARα KO (orange points) mouse hepatocytes at 24 h. (A) Accumulation plots of median BMCs for significantly enriched pathways (Fisher’s exact 2-tail <0.1). The top 6 most significantly enriched signaling pathways among concentration-responsive probes for HFPO-DA are annotated by color-coded shapes. (B) Range plots (median BMCL, median BMC, median BMCU) for the top 6 most significantly enriched signaling pathways (solid lines and circles) in WT and/or PPARα KO hepatocytes treated with HFPO-DA at 24 h. Mitochondrial fatty acid β-oxidation and sensory perception signaling pathways were also significantly enriched in PPARα KO and WT hepatocytes, respectively (indicated by dashed lines and circles), but were not among the top 6 most significan.

Similar to PPARα target genes, median BMCs for PPARα-related signaling pathways (ie, fatty acid metabolism/β-oxidation) were also approximately 10-fold higher in HFPO-DA-treated PPARα KO hepatocytes compared with WT counterparts at 24 h (Figure 8; Supplementary File 6). For example, out of the top 6 most significantly enriched signaling pathways among concentration-responsive genes in HFPO-DA-treated WT and PPARα KO hepatocytes at 24 h, most signaling pathways were related to fatty acid metabolism or peroxisomal proteins, with 5 out of the 6 pathways shared between genotypes. From these top significantly enriched pathways, median BMCs ranged from 3.3 to 4.6 µM and 31 to 32 µM for HFPO-DA-treated WT and PPARα KO hepatocytes, respectively (Figure 8). This comparison of signaling pathway enrichment among concentration-responsive genes between HFPO-DA-treated WT and PPARα KO hepatocytes was not possible at the 12- or 72-h timepoints due to the low number of concentration-responsive genes in WT and/or PPARα KO hepatocytes. Among concentration-responsive genes in GW7647-treated hepatocytes at 24 h, there were no significantly enriched (ie, Fisher’s exact 2-tail <0.1) signaling pathways in WT hepatocytes, however, the top most significantly enriched pathways in PPARα KO hepatocytes were also related to fatty acid metabolism or peroxisomal proteins and had median BMCs greater than the second highest concentration tested for GW7647, ranging from approximately 2.2 to approximately 3.8 µM (Supplementary File 6). In contrast, median BMCs for significantly enriched signaling pathways in rosiglitazone, acetaminophen, or d-galactosamine-treated hepatocytes were similar between genotypes (Supplementary File 6).

Overall, consistent with results from gene set enrichment analyses of DEGs using the hypergeometric test, PPARα-related signaling pathways were among the most significantly enriched pathways for concentration-responsive genes in both WT and PPARα KO hepatocytes treated with HFPO-DA or GW7647. However, PPARα target genes and related signaling pathways had approximately 10-fold higher BMCs and median BMCs, respectively, in HFPO-DA and GW7647-treated PPARα KO hepatocytes compared with WT counterparts. These findings coincide with the observed temporal delay of transcriptomic responses in HFPO-DA and GW7647-treated PPARα KO hepatocytes.

## Discussion

Whole transcriptomic analyses of primary WT and PPARα KO mouse hepatocytes demonstrated nearly identical transcriptomic signaling pathways and predicted upstream regulators in both hepatocyte genotypes treated with the short-chain PFAS, HFPO-DA, or the established PPARα agonist, GW7647. Pathways related to fatty acid metabolism and PPAR signaling were among the most significantly enriched for both of these chemicals. However, responses in PPARα KO hepatocytes were weaker, and exhibited a distinct temporal and concentration-dependent delay for both chemicals that did not occur in WT hepatocytes. Evidence for this delay was based on a low number of DEPs and general lack of enriched gene sets at 12 h, as well as approximately 10-fold difference between BMCs for PPARα target genes and associated pathways in WT and PPARα KO hepatocytes at 24 and 72 h. Conversely, this delay was not observed in PPARα KO hepatocytes treated with the established PPARγ agonist, rosiglitazone, or known cytotoxic agents, acetaminophen or d-galactosamine.

The delayed but similar responses in PPARα KO hepatocytes treated with HFPO-DA or GW7674 to that observed in WT hepatocytes suggest evidence for compensatory mechanisms activated in lieu of PPARα. Several transcription factors, in addition to PPARα, control fatty acid homeostasis in the liver including CAR (constitutive androstane receptor), PXR (pregnane X receptor), HNF4α (hepatocyte nuclear factor 4 alpha), and PPARδ/γ (Hernandez *et al.*, 2009; Wang *et al.*, 2020). HNF4α regulates both CAR and PPARα with the help of the coactivator, PGC-1α (peroxisome proliferator-activated receptor-gamma coactivator alpha) (Hernandez *et al.*, 2009; Kasano-Camones *et al.*, 2023). Under normal conditions, PPARα and CAR also compete for PGC-1α to activate or suppress the beta-oxidation of fatty acids, respectively. However, under certain conditions such as xenobiotic exposure in a hepatic model with increased lipids (eg, high-fat diet rodent model), CAR, and to a lesser extent PXR, can be activated to increase fatty acid metabolism (Finn *et al.*, 2009; Hernandez *et al.*, 2009). Similar to observations in high-fat diet rodent models, hepatic lipid accumulation also occurred in PPARα KO mice treated with PPARα agonists, WY-14,643, or clofibrate (Lee *et al.*, 1995). In the current study, lipid metabolizing CYPs, including those regulated by CAR or PXR, were activated at similar or slightly lower concentrations than PPARα target genes in PPARα KO hepatocytes treated with HFPO-DA or GW7647, providing some support for the induction of compensatory homeostatic mechanisms. However, it is unknown whether this induction was via direct (despite low affinity) and/or indirect (eg, crosstalk) receptor activation.

In contrast to the PPARα-activating compounds (HFPO-DA and GW7647), the PPARγ agonist rosiglitazone increased enrichment of fatty acid metabolism-related pathways at 12 h in PPARα KO hepatocytes but not in WT hepatocytes. Although PPARγ is expressed at low levels in the liver, hepatic activation of PPARγ promotes energy storage through increased lipogenesis, whereas PPARα activation promotes the release of energy via fatty acid oxidation and thermogenesis (Wang *et al.*, 2020). Given these competing roles of energy homeostasis in the liver, it is conceivable that in the absence of PPARα, rosiglitazone is more active in PPARα KO hepatocytes and induces a greater transcriptomic response than in WT hepatocytes. Moreover, unlike WT hepatocytes, where pathway-level responses for HFPO-DA, GW7647, and rosiglitazone-treated hepatocytes were similar between HFPO-DA and GW7647-treated hepatocytes but different from rosiglitazone-treated hepatocytes, greater similarity was observed across pathway-level responses in rosiglitazone, HFPO-DA and GW7647-treated PPARα KO hepatocytes, especially at higher concentrations and later timepoints. Despite being potent activators of human, rat, and mouse PPARα (Behr *et al.*, 2020; Nielsen *et al.*, 2022), HFPO-DA and GW7647 can also activate PPARγ in transactivation assays, albeit with an approximately 100-fold (or more) lower potency compared with PPARα (Behr *et al.*, 2020; Brown *et al.*, 2001; Evans *et al.*, 2022). Thus, in the absence of PPARα, at high concentrations and longer exposure durations, PPARγ might be activated by HFPO-DA and GW7647. However, as described below, due to toxicokinetic differences between *in vitro* and *in vivo* experimental systems, compensatory responses observed in PPARα KO hepatocytes treated with HFPO-DA or GW7647 are not anticipated *in vivo*. In contrast to results for rosiglitazone, no overlap in pathway enrichment was observed between HFPO-DA-treated hepatocytes and hepatocytes treated with cytotoxic agents, regardless of hepatocyte genotype, providing further support that HFPO-DA does not act through a cytotoxic MOA (Heintz *et al.*, 2023).

In PPARα KO hepatocytes, both GW7647 and HFPO-DA exhibited transient low-dose effects at 12 and 24 h, respectively. HFPO-DA appeared to induce changes related to protein degradation, whereas GW7647 appeared to affect mitochondrial fatty acid β-oxidation. Whether these low-dose changes are the result of experimental factors (eg, handling issues), transcriptomic “noise,” or true biological responses is unclear. Interestingly, PPARs and other nuclear receptors are intricately controlled by the ubiquitin proteosome system (UPS), and each has unique responses to ligands—including stabilization of function, targeting for degradation, or, in the case of PPARα, transient stabilization and subsequent degradation (Genini *et al.*, 2008). In mice, exposure to clofibrate for 2 weeks has been shown to decrease expression of murine double minute 2 (*Mdm2*), an E3 ubiquitin ligase that binds to and regulates PPARα, in WT mice but not PPARα KO mice indicating a potential negative feedback loop to control PPARα activation in the presence of a ligand (Gopinathan *et al.*, 2009). These complex interactions between ligands, receptors, and coactivators/regulators (eg, Mdm2) might partially explain the apparent nonmonotonic (and different) transcriptomic changes observed at low and high doses, which might be more apparent in the absence of PPARα.

Interestingly, available *in vivo* studies in PPARα KO mice have virtually no transcriptomic response (eg, few to no significant DEGs and no pathway-level response) following subacute or chronic exposures to established PPARα agonists including WY-14,643 or GW7647 (Foreman *et al.*, 2021; Rakhshandehroo *et al.*, 2007). In addition, transcriptomic responses for HFPO-DA have also been examined in PPARα KO mice administered a high-fat diet in combination with a low dose (0.3 mg/kg-bw/day) of HFPO-DA for 20 weeks. Although there was no negative control for the high-fat diet in this study, the authors determined that effects of HFPO-DA exposure on hepatic gene expression were dependent on PPARα, due to “no significant gene regulation” by HFPO-DA in PPARα KO mice (Attema *et al.*, 2022). These results suggest that the delayed transcriptomic response observed *in vitro* for HFPO-DA and GW7647-treated PPARα KO hepatocytes may be a consequence of the experimental system used. *In vivo*, chemical toxicokinetics can greatly affect the amount and duration of exposure in a specific tissue or cell type; whereas *in vitro*, the exposure is typically more controlled and constant, and when under stable conditions (eg, no chemical precipitation or volatilization) generally only differs if the test chemical is metabolized. For example, HFPO-DA is absorbed and rapidly eliminated in the urine of rats, mice, and monkeys, with an elimination half-life on the order of hours. In addition, HFPO-DA is not metabolized by rodent liver *in vitro* or *in vivo* (Gannon *et al.*, 2016). To our knowledge, the toxicokinetics of GW7647 have not been investigated, however, the elimination half-life of GW7647 is also expected to be on the order of hours, similar to PPARα agonist drugs (eg, fenofibrate; Chapman, 1987). Thus, the delayed compensatory responses observed *in vitro* for HFPO-DA and GW7647 in PPARα KO hepatocytes are not anticipated *in vivo* in PPARα KO mice due to the rapid elimination of these chemicals.

The *in vitro* system used in this study is best suited for examining early key events or molecular initiating events in the HFPO-DA MOA. The lack of concordance between HFPO-DA and cytotoxic agents in both WT and PPARα KO hepatocytes indicates that liver effects observed in mice following HFPO-DA exposure are not the result of a cytotoxic MOA. With regard to support for a PPARα MOA, the *in vitro* system herein is primarily capable of addressing Key Event 1 (PPARα activation). An assessment of downstream key events (ie, alteration of cell growth pathways, perturbation of cell growth and survival, and selective clonal expansion of preneoplastic foci cells) is not feasible in cultured hepatocytes lacking nonparenchymal cells (eg, Kupffer cells) that facilitate cell proliferation (McMullen *et al.*, 2020; Szalowska *et al.*, 2014). Because the influence of PPARα genotype on alteration of cell growth and survival cannot be readily assessed *in vitro*, *in vivo* studies are currently underway to further address Key Event 1 and subsequent key events in WT and PPARα KO mice. In addition to transcriptomic analyses, these *in vivo* studies will include examination of phenotypic responses (eg, histopathological changes, serum liver enzymes) to further inform MOA.

In summary, the study design herein allows for the investigation of PPARα-dependent and independent mechanisms across several chemicals, concentrations, and timepoints. The similar transcriptomic signaling patterns between HFPO-DA and an established PPARα agonist (GW7647) in both WT and PPARα KO hepatocytes support a shared MOA for both chemicals *in vitro* and presumably *in vivo*. These findings also provide mechanistic insight as to what happens in PPARα KO mouse hepatocytes when treated with PPARα and PPARγ activators. PPAR signaling and fatty acid metabolism-related pathways are mediated more efficiently in WT hepatocytes treated with HFPO-DA and GW7647, however, similar transcriptomic signaling potentially mediated by compensatory responses was also observed in the absence of PPARα, albeit weaker and delayed. In contrast, rosiglitazone, appears more active in the absence of the PPARα, likely due to the competing roles between PPARα and PPARγ in the liver for energy homeostasis.

## Supplementary Material

kfae045_Supplementary_Data

## Data Availability

RNA sequencing data are publicly available at NCBI’s Gene Expression Omnibus (https://www.ncbi.nlm.nih.gov/geo/) (GEO series accession number GSE248251). All Supplementary Files 1–6 are available at DOI: 10.5061/dryad.pc866t1wp.

## Footnotes

1 Note: A single specific genetic background strain for B6;129S4-Ppara^tm1Gonz^/J does not exist, however, either B6129SF2/J or C57BL/6J strains have been determined to be appropriate background strains based on what is known regarding the B6;129S4-Ppara^tm1Gonz^/J genome. In the companion publication, transcriptomic responses for each chemical investigated were consistent (especially for PPARα activators, HFPO-DA and GW7647) across hepatocytes from different mouse strains (CD-1 and B6129SF2/J) and rodent species (ie, rats), indicating that the genetic differences between rodent strains/species did not impact the overall transcriptomic response/MOA for the chemicals investigated.
