# Supplementary material for: Comparison of transcriptomic profiles between HFPO-DA and prototypical PPARα, PPARγ, and cytotoxic agents in wild-type and PPARα knockout mouse hepatocytes
Source: Toxicol Sci. 2024 Apr 4;200(1):183–98. doi: 10.1093/toxsci/kfae045 (PMC11199908; doi:10.1093/toxsci/kfae045)
Supplement: kfae045_Supplementary_Data [file kfae045_supplementary_data.docx]

**Supplementary Figures & Tables**

**Figure S1. LDH release measured at 12, 24 and 72 h in control and treated B6129SF2/J and PPARα KO mouse hepatocytes.**

**Figure S2. Principle component analysis plot of WT (A) and PPARα KO (B) mouse hepatocyte samples.** The chemical treatment group of each sample is indicated by color-coded shapes, with shape and color indicating chemical treatment and size indicating concentration level (see legend).

**Figure S3. Principle component analysis and scree plots of WT (A) and PPARα KO (B) mouse hepatocyte samples by timepoint.** The chemical treatment group of each sample is indicated by color-coded shapes, with shape and color indicating chemical treatment and size indicating concentration level (see legend).

**Figure S4. Principle component analysis plots of WT (A) and PPARα KO (B) mouse hepatocyte samples by timepoint with cytotoxic agents (acetaminophen and d-galactosamine) removed.** The chemical treatment group of each sample is indicated by color-coded shapes, with shape and color indicating chemical treatment and size indicating concentration level (see legend).

**Figure S5. Hierarchical cluster analyses of** **B6129SF2/J mouse (A) and PPARα KO mouse (B) hepatocyte samples as well as samples from both mouse genotypes together (C).** Unsupervised clustering according to Euclidean distances was demonstrated by the ordering of individual hepatocyte samples, with distances depicted by dendrograms. Color and grayscale identifiers are assigned to each sample across the bottom of the hierarchical clustering figure according to mouse genotype (turquoise and brown for B6129SF2/J and PPARα KO mouse, respectively), timepoint (grayscale increasing in darkness with increasing exposure duration), chemical treatment (light gray for solvent controls, green, blue, red, orange and purple for HFPO-DA, GW7674, rosiglitazone, acetaminophen and d-galactosamine, respectively), and concentration (grayscale increasing in darkness with increasing concentration). Methods used to perform hierarchical clustering across hepatocyte samples were consistent with methods used in the companion publication. Mouse probes were converted to human gene names using the R package biomaRt (v2.56.1) based on the Ensembl genome database. For genes for which multiple probes were used to measure expression, the probe with the highest mean sequencing count across all samples was used in hierarchical cluster analysis. Normalized gene expression data for each mouse genotype were collated, and whole transcriptome data were subset using the National Toxicology Program’s S1500+ gene set list for humans (Mav et al. 2018). Samples were clustered using normalized expression level per sample per probe, which was estimated by the distance of each individual sample from the mean expression level for that probe across all samples. R packages dendextend (v1.17.1), stringr (v1.5.1) and stats (base version) were used to conduct the analyses in R (v4.3.1).

**Figure S6. Number of significantly downregulated DEPs (FDR < 10% and enriched gene sets (FDR < 5%) for each chemical tested (relative to controls) in WT and PPARα KO mouse hepatocytes across 12, 24 and 72 h.** Each row represents a different chemical and each column represents a different test concentration, with concentrations increasing from left to right. An “*” indicates that cytotoxicity was observed at this concentration and timepoint. An “#” indicates that samples from this concentration and timepoint did not undergo transcriptomic analyses due to low sequencing quality.

**Figure S7. ToxPi visualizations of upregulated gene set aggregation results for WT and PPARα KO mouse hepatocytes using external scaling approach.** Significant (FDR<5%) upregulated gene sets from hypergeometric gene set enrichment analysis containing genes known to interact with HFPO-DA and/or positive controls were aggregated as described in the Methods section. The size of a ToxPi wedge for a given gene reflects the significance and number of enriched gene sets containing that gene within a specific chemical treatment group and timepoint that is scaled in respect to the same gene wedge across ToxPis for different chemical treatment groups/timepoints (i.e., external scaling). An “*” indicates that cytotoxicity was observed at this concentration and timepoint. An empty ToxPi indicates that none of the targeted gene sets were enriched significantly, and “NA” means that samples from this concentration and timepoint did not undergo transcriptomic analyses due to low sequencing quality.

**Supplementary Figure S8. Top 20 predicted upstream regulators in WT and PPAR⍺ KO hepatocytes at 12 h using IPA upstream analyses.** Each column represents a different test concentration, with concentrations increasing from left to right for each chemical. Orange indicates predicted activation, and blue indicates predicted inhibition; the intensity of each color increases with the absolute z-score. Columns with no z-score prediction indicate chemical treatment groups with a low number DEGs and upstream regulator predictions were not able to be estimated.

**Supplementary Figure S9. Top 20 predicted upstream regulators in WT and PPAR⍺ KO hepatocytes at 24 h using IPA upstream analyses.** Each column represents a different test concentration, with concentrations increasing from left to right for each chemical. Orange indicates predicted activation, and blue indicates predicted inhibition; the intensity of each color increases with the absolute z-score. Columns with no z-score prediction indicate chemical treatment groups with a low number DEGs and upstream regulator predictions were not able to be estimated.

**Supplementary Figure S10. Top 20 predicted upstream regulators in WT and PPAR⍺ KO hepatocytes at 72 h using IPA upstream analyses.** Each column represents a different test concentration, with concentrations increasing from left to right for each chemical. Orange indicates predicted activation, and blue indicates predicted inhibition; the intensity of each color increases with the absolute z-score. Columns with no z-score prediction indicate chemical treatment groups with a low number DEGs and upstream regulator predictions were not able to be estimated.

**Figure S11. Accumulation plots of best BMCs among significant concentration-responsive probes (best fit *p*-value ≥ 0.1) in WT and PPARα KO mouse hepatocytes at 12 h.** Concentration-responsive probes are indicated by blue and orange points for WT and PPARα KO mouse hepatocytes, respectively. Best BMCs of PPARα target genes and lipid metabolizing cytochrome P450’s (CYPs) are annotated by color-coded shapes. The PPARα target genes consist of 12 genes identified as HFPO-DA and/or GW7647-interacting genes highlighted in the ToxPi key in Figure 3 and S3.

**Figure S12. Accumulation plots of best BMCs among significant concentration-responsive probes (best fit *p*-value ≥ 0.1) in WT and PPARα KO mouse hepatocytes at 72 h.** Concentration-responsive probes are indicated by blue and orange points for WT and PPARα KO mouse hepatocytes, respectively. Best BMCs of PPARα target genes and lipid metabolizing cytochrome P450’s (CYPs) are annotated by color-coded shapes. The PPARα target genes consist of 12 genes identified as HFPO-DA and/or GW7647-interacting genes highlighted in the ToxPi key in Figure 3 and S3.

**Figure S13. Accumulation plots of best BMCs among significant up- or downregulated concentration-responsive probes (best fit *p*-value ≥ 0.1) in WT and PPARα KO mouse hepatocytes at 24 h.** Upregulated concentration-responsive probes are indicated by light blue and orange points for WT and PPARα KO mouse hepatocytes, respectively. Downregulated concentration-responsive probes are indicated by dark blue and dark orange points for WT and PPARα KO mouse hepatocytes, respectively. Best BMCs of PPARα target genes and lipid metabolizing cytochrome P450’s (CYPs) are annotated by color-coded shapes. The PPARα target genes consist of 12 genes identified as HFPO-DA and/or GW7647-interacting genes highlighted in the ToxPi key in Figure 3 and S3.

**Table S1. Number of samples remaining in each treatment group following removal of samples that failed sequencing quality criteria (see Supplementary File S1 Table 2).**

* Treatment group was removed from transcriptomic analyses.
